# NEIGHBORHOOD SOCIAL COHESION, RESILIENCE, AND WELL-BEING AMONG CHINESE OLDER ADULTS IN HONOLULU, HAWAII

**DOI:** 10.1093/geroni/igz038.2187

**Published:** 2019-11-08

**Authors:** Wei Zhang, Sizhe Liu, Keqing Zhang, Bei Wu

**Affiliations:** 1 University of Hawaii at Manoa, Honolulu, Hawaii, United States; 2 New York University, New York, New York, United States

## Abstract

Few studies have examined the association of social environment and well-being among Chinese older adults, the fastest growing aging population across all racial/ethnic groups in the U.S. To address this gap, the current study aims to examine the associations of neighborhood social cohesion with psychological distress and life satisfaction as well as the mediating role of resilience and the moderating roles of gender and place of birth using data collected among 430 Chinese older adults in Honolulu. Results show that neighborhood cohesion was significantly associated with both distress and life satisfaction, with resilience being a significant mediator. The association between neighborhood cohesion and distress was moderated by birth place such that the protecting effects of neighborhood cohesion on distress were only salient for the U.S.-born. Our findings indicate the importance of a cohesive social environment in shaping well-being of U.S. Chinese older adults, the U.S.-born in particular, living in Hawai’i.

